# Physiological Criteria Are Useful for the Diagnosis of Idiopathic Pleuroparenchymal Fibroelastosis

**DOI:** 10.3390/jcm9113761

**Published:** 2020-11-22

**Authors:** Takato Ikeda, Yoshiaki Kinoshita, Yusuke Ueda, Tomoya Sasaki, Hisako Kushima, Hiroshi Ishii

**Affiliations:** Department of Respiratory Medicine, Fukuoka University Chikushi Hospital, Fukuoka 818-8502, Japan; t-ikeda@fukuoka-u.ac.jp (T.I.); torreofyu@yahoo.co.jp (Y.U.); 1982tomo@fukuoka-u.ac.jp (T.S.); hkushi@fukuoka-u.ac.jp (H.K.); hishii@fukuoka-u.ac.jp (H.I.)

**Keywords:** residual volume, flat chest index, body mass index, diagnostic criteria, pleuroparenchymal fibroelastosis

## Abstract

Background: Diagnostic criteria of idiopathic pleuroparenchymal fibroelastosis (IPPFE) were recently proposed, including physiological criteria of the body mass index (BMI) and percentage of the predicted values of residual volume (RV)/total lung capacity (TLC) (RV/TLC %pred.). The aim of this study was to evaluate (i) whether the physiologic criteria are useful for the diagnosis and (ii) whether the flat chest index, defined as the ratio of the anteroposterior diameter to the transverse diameter of the thoracic cage, could be an alternative parameter to RV/TLC %pred. Methods: We selected consecutive IPPFE patients and idiopathic pulmonary fibrosis (IPF) patients. We examined the diagnostic sensitivity and specificity of the physiological criteria and flat chest index for differentiating IPPFE patients from IPF patients. Results: This study included 37 IPPFE patients and 89 IPF patients. The physiological criteria distinguished IPPFE patients from IPF patients with a sensitivity of 78.6% and specificity of 88.0%. The combination of the flat chest index and BMI was also effective for differentiation (sensitivity of 82.1% and specificity of 89.3%). Conclusion: We verified the good performance of the physiologic criteria in a different cohort. When the RV/TLC is not measured, using the flat chest index instead of RV/TLC %pred. may be reasonable.

## 1. Introduction

Idiopathic pleuroparenchymal fibroelastosis (IPPFE) is a chronic and progressive interstitial pneumonia that predominantly affects the upper lung fields [1,2,3]. Pathologically, IPPFE is characterized by fibroelastosis in subpleural lung tissue and collagenous fibrosis in the pleura [1,2,3]. In 2013, IPPFE was included in the current classification of idiopathic interstitial pneumonias (IIPs) as a rare subtype [3]. The subsequent accumulation of cases has led to the identification of various clinical features of IPPFE, including emaciation [1,4,5,6,7,8,9,10], a flattened thoracic cage [1,11,12,13,14,15], disproportionately decreased forced vital capacity (FVC), and increased percentage of the predicted values of residual volume (RV)/total lung capacity (TLC) (RV/TLC %pred.) [4,11,16,17,18,19,20,21]. These clinical advances have resulted in the proposal of several diagnostic criteria of IPPFE [13,20,22].

Watanabe et al. [13] recently proposed diagnostic criteria of IPPFE that describe four categories based on the clinical symptoms, radiology, histology, and physiology: “definite IPPFE”, “radiologically and physiologically probable IPPFE”, “radiologically probable IPPFE”, and “radiologically possible IPPFE”. A noteworthy feature of the diagnostic criteria is that two physiological parameters—the body mass index (BMI) and RV/TLC %pred.—are applied to the category of radiologically and physiologically probable IPPFE. The physiological criteria can clinically differentiate IPPFE patients from idiopathic pulmonary fibrosis (IPF) patients, a sometimes difficult task. In addition, performing a tissue biopsy for the diagnosis of PPFE is discouraged because PPFE is occasionally complicated by refractory pneumothorax [1,2,5,6,20,22]. Therefore, the physiologic criteria are considered essential to the diagnosis of IPPFE.

Watanabe et al. [13] showed that the physiological criteria successfully discriminated IPPFE patients from IPF patients with a sensitivity of 87.8% and a specificity of 83.5%. However, the significance of the physiological criteria for the diagnosis of IPPFE has not yet been validated.

Meanwhile, lung capacities of RV and TLC using plethysmography are evaluated less frequently than the spirometric parameters of FVC or forced expiratory volume in 1 second (FEV_1_). In addition, some IPPFE patients cannot have their RV/TLC measured due to an impaired respiratory function. However, IPPFE patients without an RV/TLC measurement cannot be assessed for whether or not they meet the physiological criteria of IPPFE. Therefore, alternative parameters are necessary for suspected IPPFE cases in which the lung capacities are not measured. The radiological characteristics of IPPFE patients include an abnormally narrow anterior-posterior thoracic diameter, known as platythorax [1,11,12,13,14,15]. Therefore, we considered that it may be reasonable to use the flat chest index defined as the ratio of the anteroposterior diameter to the transverse diameter of the thoracic cage on computed tomography, when the RV/TLC is not measured.

The present study was therefore designed to address these two-fold objectives: (i) whether the physiologic criteria of IPPFE are useful for the diagnosis and (ii) whether the flat chest index could be an alternative physiologic parameter to RV/TLC %pred.

## 2. Materials and Methods

### 2.1. Subjects

We retrospectively reviewed the medical records of the Department of Respiratory Medicine at Fukuoka University Chikushi Hospital from 2011 to 2020. Consecutive patients with suspected IPPFE who had upper-lobe dominant fibrosis were collected. Three observers (T.I., Y.K., and H.I.) reevaluated and excluded patients who had been diagnosed with interstitial lung disease (ILD) other than IPPFE by clinical, radiological, or histological examinations (if available). Disagreements were resolved by consensus. The diagnosis of IPPFE (definite IPPFE, radiologically and physiologically probable IPPFE, and radiologically probable IPPFE) was made according to the criteria proposed by Watanabe et al. [13]. Patients who had been classified as having radiologically possible IPPFE were excluded from the study because radiologically possible IPPFE might include apical cap fibrosis other than the early stages of IPPFE [13].

Consecutive patients with IPF were collected for the comparison with IPPFE. The diagnosis of IPF was made based on the 2018 American Thoracic Society/European Respiratory Society/Japanese Respiratory Society/Latin American Thoracic Association clinical practice guidelines [23]. The Fukuoka University Medical Ethics Review Committee approved the study protocol and waived the requirement for informed consent (approval number: C20-09-002).

### 2.2. Clinical Characteristics and Respiratory Function Data

Clinical characteristics, including the age, gender, BMI, smoking status, and serum Krebs von den Lungen-6 (KL-6) concentration, were obtained from the medical records. The respiratory function parameters examined were the FVC, FEV_1_, functional residual capacity (FRC), RV, TLC, and diffusion capacity of the lung for carbon monoxide (DLco). The respiratory function parameters were measured as previously described [24,25,26].

The date of the diagnosis was defined as the date of the first radiological evaluation by computed tomography (CT). We decided whether or not the enrolled patients meet the physiological criteria for IPPFE: (a) RV/TLC %pred. ≥ 115% or b) RV/TLC %pred. ≥ 80% and BMI ≤ 20 kg/m^2^ [12]. To do so, the respiratory function parameters obtained on the date closest to the diagnosis were assessed. We then compared the clinical characteristics of IPPFE patients who met the physiological criteria at the time of the diagnosis and those who did not. If the respiratory function parameters had been measured multiple times during the observation periods of IPPFE, we also assessed whether or not each parameter obtained after the diagnosis met the physiological criteria.

### 2.3. The Flat Chest Index

**Figure 1:**
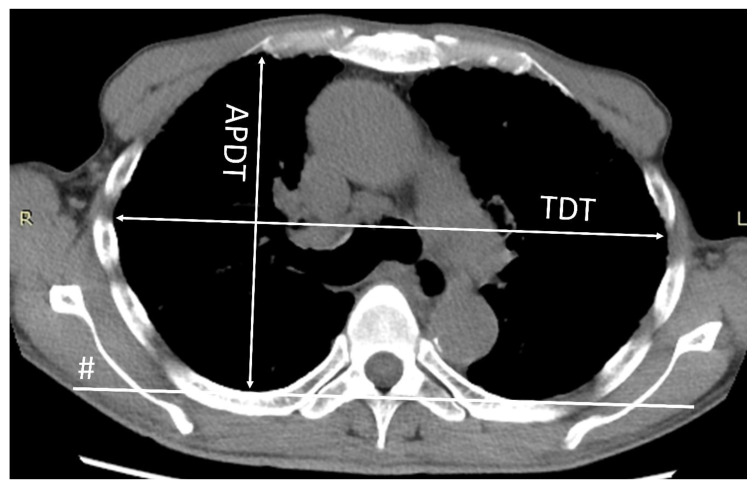
The transverse diameter of the thoracic cage (TDT) was determined as the longest transverse diameter of the thoracic cage measured parallel to a line (#) that runs along the rearmost points of the bilateral 6th ribs in the horizontal section of the CT scan. The anteroposterior diameter of the thoracic cage (APDT) was determined as the longest distance of the anteroposterior dimension of the thoracic cage measured perpendicular to the line along the rearmost points of the sixth thoracic vertebra (line #).

The flat chest index was defined as the ratio of the anteroposterior diameter to the transverse diameter of the thoracic cage at the level of the sixth thoracic vertebra on CT, as described previously (Figure 1) [15]. The flat chest index was calculated using the CT scan obtained at the time of the diagnosis. The correlation between the flat chest index and RV/TLC %pred. in IPPFE patients was then analyzed.

### 2.4. Diagnostic Sensitivity and Specificity of the Physiological Criteria

Among patients with at least one measurement of RV/TLC, we evaluated the diagnostic sensitivity and specificity of the physiological criteria for differentiating IPPFE patients from IPF patients. The areas under the curve (AUCs) of these parameters for discriminating IPPFE from IPF were compared among parameters or with the data described by Watanabe et al [13].

### 2.5. Statistical Analyses

Continuous data are presented as the mean ± standard deviation or median (interquartile range) of the groups. Fisher’s exact test was used to compare categorical variables. Differences between groups were assessed using Student’s *t*-test for unpaired data and the Mann–Whitney U test for continuous variables. The AUC of each parameter was determined by a receiver operating characteristic (ROC) curve analysis. Spearman’s correlation coefficients were used to assess the association between variables. Kappa statistics were used to assess the agreement of the results between each physiological parameter. A *p*-value < 0.05 was considered to indicate a statistically significant difference. All statistical analyses were performed using R software program (version 3.2.2.2: R Foundation for Statistical Computing, Vienna, Austria).

## 3. Results

### 3.1. Study Patients

**Figure 2:**
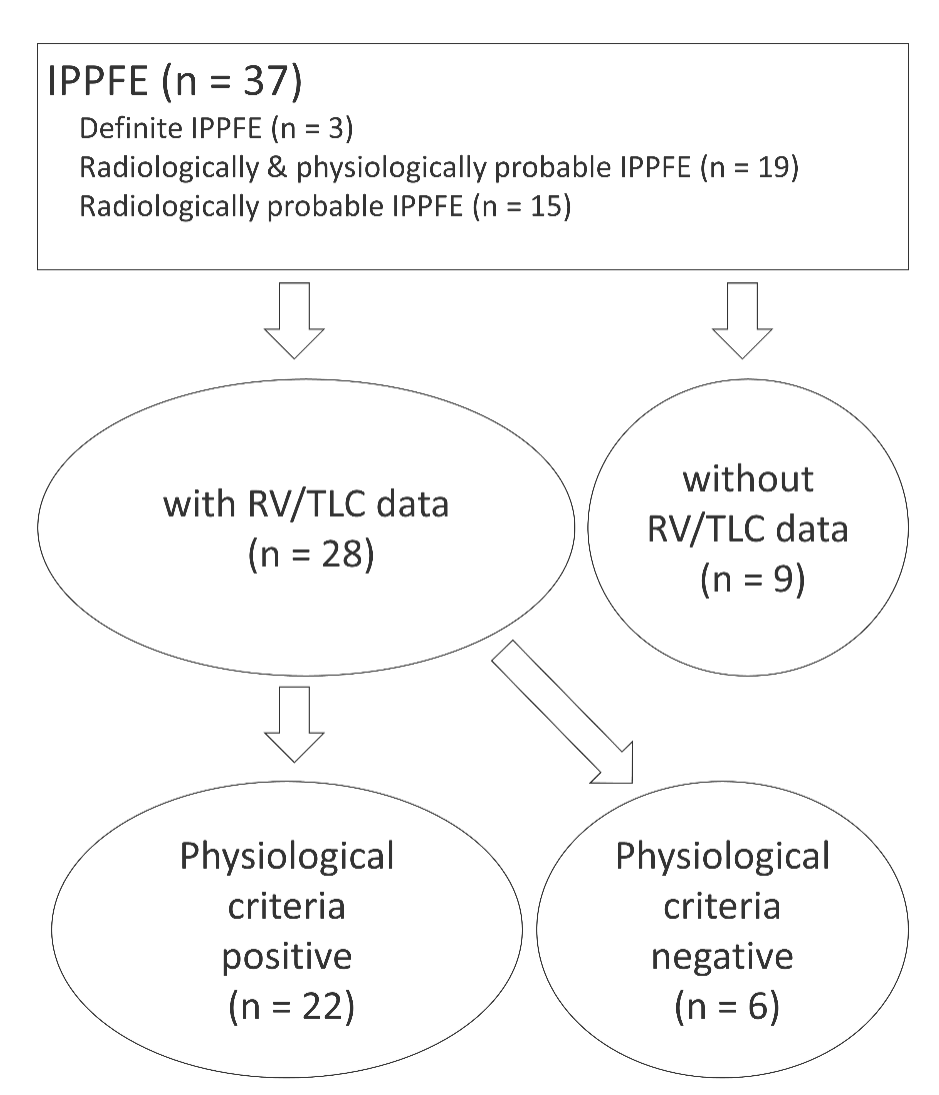
A schematic diagram of the enrolled idiopathic pleuroparenchymal fibroelastosis (IPPFE) patients. RV, residual volume; TLC, total lung capacity.

Sixty-seven patients were clinically suspected of having IPPFE, but 30 were excluded because they had been diagnosed with radiologically possible IPPFE (*n* = 14), unclassifiable IIP (*n* = 5), nontuberculous mycobacterial infection (*n* = 5), secondary PPFE (*n* = 3), chronic hypersensitivity pneumonia (*n* = 1), sarcoidosis (*n* = 1), or postoperative unilateral upper lung field pulmonary fibrosis (*n* = 1) [27]. A total of 37 patients with IPPFE were eligible for the study and classified as having definite IPPFE (*n* = 3), radiologically and physiologically probable IPPFE (*n* = 19), or radiologically probable IPPFE (*n* = 15). A schematic diagram of the enrolled IPPFE patients is shown in Figure 2.

For the comparison with IPPFE, 89 consecutive patients with IPF were selected. Radiologically, IPF patients were classified as having usual interstitial pneumonia (UIP) (*n* = 71) or probable UIP (*n* = 18) based on the guideline [23]. Eight IPF patients underwent a surgical lung biopsy and were histologically classified as UIP (*n* = 4), probable UIP (*n* = 3), or indeterminate for UIP (*n* = 1).

### 3.2. Patients’ Characteristics

Patients’ characteristics are summarized in Table 1. IPPFE patients had significantly fewer pack-years of smoking, a lower age, lower prevalence of male gender, lower BMI, lower flat chest index, and lower serum KL-6 levels than IPF patients. Respiratory function testing showed that IPPFE patients had significantly lower values for FVC and FEV_1_/FVC and higher values for RV and RV/TLC %pred. than IPF patients.

### 3.3. Diagnostic Sensitivity and Specificity of the Physiological Criteria

Twenty-eight of the 37 IPPFE patients and 75 of the 89 IPF patients had undergone measurement of the RV/TLC at least once. Among them, 22 of the 28 IPPFE patients and nine of the 75 IPF patients met the physiological criteria of IPPFE. The diagnostic sensitivity of the physiological criteria for discriminating IPPFE patients from IPF patients was slightly lower, but the specificity was equivalent to those in the study by Watanabe et al. (sensitivity of 78.6% vs. 87.8% and specificity of 88.0% vs. 83.5%) (Table 2).

**Figure 3:**
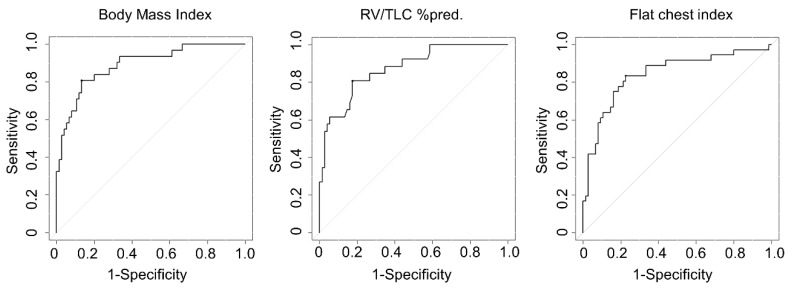
The results of the receiver operating characteristic (ROC) curve analyses. The area under curves of the Body Mass Index (0.875) and RV/TLC %pred. (0.878) were slightly higher than that of the flat chest index (0.837).

An ROC curve analysis showed that the AUCs of the BMI (0.875) and RV/TLC %pred. (0.878) were slightly higher than that of the flat chest index (0.837) (Table 2 and Figure 3). The sensitivity and specificity of the BMI with a cut-off level of 20 were equivalent between the present study and that of Watanabe et al. (sensitivity of 75% vs. 78.1% and specificity of 88.0% vs. 82.5%, respectively). The sensitivity of the RV/TLC %pred. was slightly lower, but the specificity was higher than that in the study by Watanabe et al. (sensitivity of 64.3% vs. 75.6% and specificity of 93.0% vs. 88.7%, respectively).

### 3.4. An Alternative Physiologic Parameter for RV/TLC %pred.

**Figure 4:**
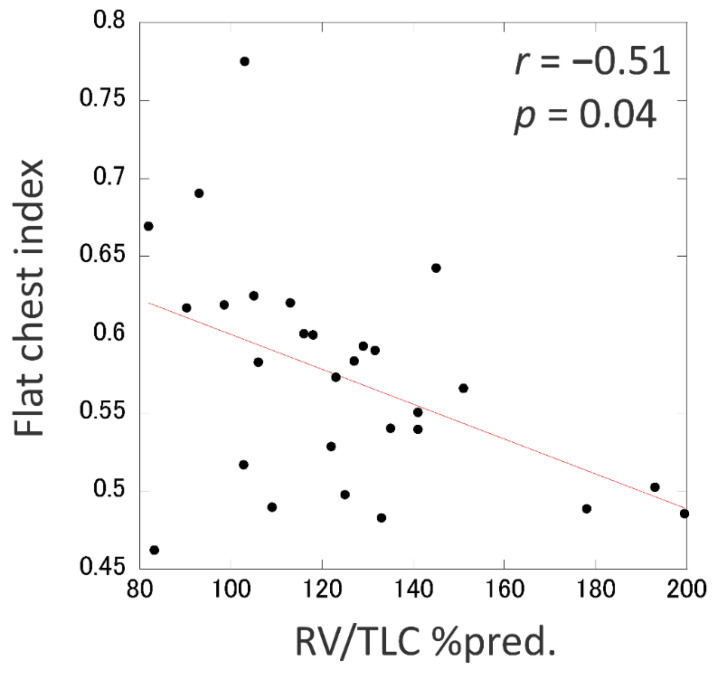
The flat chest index was inversely and significantly correlated with the percentage of the predicted value of the residual volume/total lung capacity (RV/TLC % pred.). Based on the equation of “flat chest index = −0.00111 × (RV/TLC % pred.) + 0.711” derived from a simple linear regression analysis, the cut-off values of RV/TLC %pred. (80 and 115) were considered to correspond to those of the flat chest index (0.62 and 0.58, respectively).

Nine of the 37 IPPFE patients did not have any RV/TLC data because of severe respiratory dysfunction (*n* = 4) or simply because the lung capacities were not examined (*n* = 5). To overcome the concern that these patients had no opportunity to meet the physiological criteria, we focused on the flat chest index as an alternative physiologic parameter to the RV/TLC %pred. The flat chest index was inversely and significantly correlated with the RV/TLC %pred. (*r*, −0.51; *p* = 0.04) (Figure 4). Using a simple linear regression model, the cut-off values of RV/TLC %pred. of 80 and 115 were found to correspond to flat chest indices of 0.62 and 0.58, respectively.

**Figure 5:**
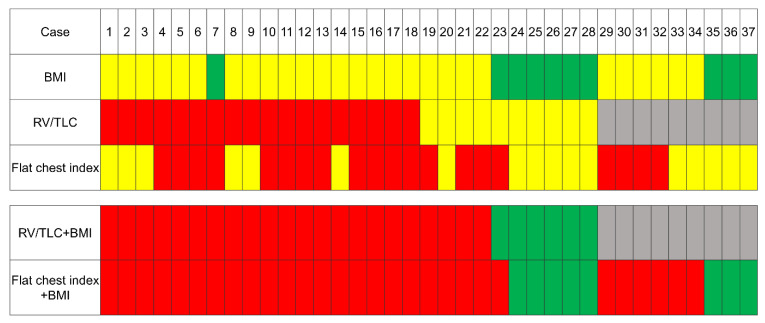
A summary of the results determined according to the cut-off values of each physiological parameter in IPPFE patients. (█) represents the results that were directly related to the diagnosis: a percentage of the predicted values of residual volume/total lung capacity (RV/TLC %pred.) ≥ 115% or a flat chest index ≤ 0.58. (█) represents the results that may lead to the diagnosis when combined: a body mass index (BMI) ≤ 20 kg/m^2^, RV/TLC %pred. ≥ 8 0% but <115%, or flat chest index > 0.58 but ≤0.62. (█) represents the results that do not lead to a diagnosis: a BMI > 20, RV/TLC %pred. < 80, or flat chest index <0.62. (█) represents no available data.

The diagnosis using a combination of the flat chest index and BMI, i.e., (a) flat chest index ≤ 0.58 or (b) >0.58 flat chest index but ≤0.62 and BMI ≤ 20, was highly concordant with the diagnosis according to the combination of the RV/TLC %pred. and BMI (kappa coefficient, 0.88) (Figure 5, case 1–28). In the present study, the diagnostic sensitivity and specificity of a combination of the flat chest index and BMI were equivalent to those obtained with the physiologic criteria (Table 2) (sensitivity of 82.1% vs. 78.6% and specificity of 89.3% vs. 88.0%, respectively). While IPPFE patients who did not have RV/TLC %pred. data could not be assessed for whether or not they met the physiological criteria, 66.7% (6/9) of them met the alternative criteria using the combination of the BMI and flat chest index (Figure 5, case 29–37).

### 3.5. Clinical Characteristics of IPPFE Patients Who Did Not Meet the Physiological Criteria

Among IPPFE patients who had at least one measurement of RV/TLC (*n* = 28), those who met the physiological criteria (*n* = 22) had a lower FVC than those who did not (*n* = 6) (58.4% (46.8–72.8%) vs. 91.8% (84.1–96.2%), *p* = 0.008) (Figure 2 and Table 3).

**Figure 6:**
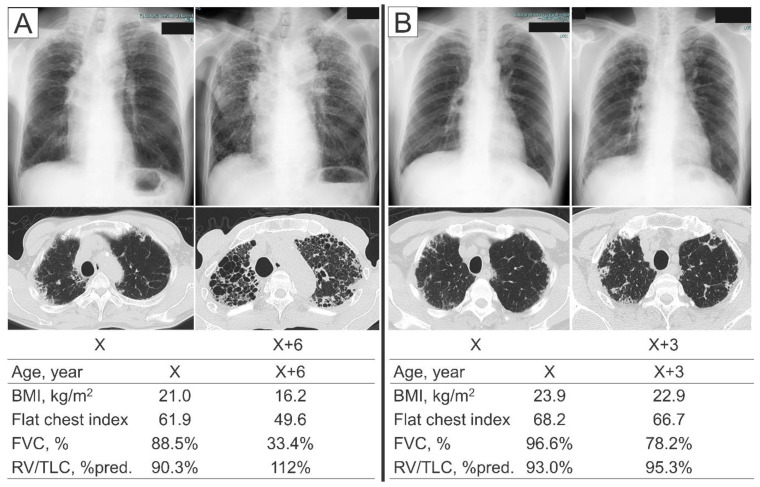
Representative IPPFE patients who did not meet the physiologic criteria at the time of the diagnosis. (**A**) A 68-year-old man who came to meet the physiological criteria during the clinical course. Although the forced vital capacity (FVC) was relatively well preserved at the time of the diagnosis, he experienced a marked decline in the FVC over the six years from the diagnosis, accompanied by decreases in the body mass index (BMI) and flat chest index and an increase in the percentage of the predicted values of residual volume/total lung capacity (RV/TLC %pred.). (**B**) A 68-year-old man who did not meet the physiological criteria during the observation period. The FVC was relatively well preserved at the time of the diagnosis as well as three years after the diagnosis. The BMI, flat chest index, and RV/TLC %pred. did not change markedly during the three years.

Among IPPFE patients who did not meet the physiologic criteria (*n* = 6), some came to meet the physiological criteria during the observation period (3/6, 50%), but others did not (3/6, 50%). Representative cases were shown in Figure 6. None of the IPPFE patients who met the physiological criteria at the time of the diagnosis deviated from the criteria during the observation period.

## 4. Discussion

In this study, the physiological criteria of IPPFE according to the combination of RV/TLC %pred. and the BMI, distinguished IPPFE patients from IPF patients with a sensitivity of 78.6% and a specificity of 88.0%. The sensitivity was slightly lower, but the specificity equaled those values reported by Watanabe et al. [13], demonstrating that the physiological criteria are valid and reproducible.

The core disease in IIPs has been and will continue to be IPF, as IPF has the highest frequency and the poorest prognosis among IIPs [23]. The history of IPPFE is relatively new compared to that of IPF, and the diagnostic criteria of IPPFE have only recently been proposed. Watanabe et al. [13] suggested that the diagnostic criteria of IPPFE should first include its clinical characteristics and then evolve into more refined and simplified criteria, as has happened with the diagnostic criteria of IPF [28,29]. One of the important clinical challenges for the diagnostic criteria of IPPFE is differentiating IPPFE patients from IPF patients [12,13,24,26]. Physiological criteria of IPPFE are essential because they can distinguish the two entities with high sensitivity and specificity [13]. In addition, if the physiologic criteria prove to be useful, there might be no clinical significance in performing a tissue biopsy for the diagnosis of PPFE at risk of pneumothorax. However, while the diagnostic criteria have been proposed, there have been no validation studies conducted in different cohorts. This is the first study to verify the good performance of the physiologic criteria of IPPFE.

A common lung function pattern in cases of IPPFE is a restrictive abnormality with a retained RV and decreased TLC [4,11,16,17,18,19,20,21]. In general, patients with simple restrictive abnormalities on respiratory function testing show a comparable decrease in the TLC and FVC [30]. However, IPPFE patients usually show complex restrictive abnormalities, a rare form of the restrictive pattern characterized by a disproportionately decreased FVC relative to the decrease in TLC with an increase in the RV/TLC %pred [4,11,16,17,18,19,20,21]. Clay et al. [30] showed that this complex restrictive abnormality is due to air trapping secondary to obstructive impairment because of airway closure at high lung volumes or to a mechanical inability to reduce the thoracic volume, as in cases of neuromuscular weakness and some chest wall disorders. The latter mechanism may be the cause of the complex restrictive abnormality in PPFE because the progressive contraction of the upper lobes surrounded by pleural fibrosis and subpleural fibroelastosis causes the thoracic cage to be flattened in IPPFE [15]. Thus, an increase in the RV/TLC %pred. is theoretically related to the decrease in the flat chest index in PPFE patients. Indeed, we detected a significant correlation between the RV/TLC %pred. and flat chest index, as previously reported in another cohort [11].

In the present study, the flat chest index was a useful indicator for distinguishing IPPFE patients from IPF patients, although the sensitivity was slightly lower than that of the RV/TLC %pred. or BMI. The diagnosis according to the combination of the flat chest index and BMI was highly concordant with the diagnosis according to the combination of the RV/TLC %pred. and BMI (kappa = 0.88) (Figure 5). In addition, 66.7% of IPPFE patients who lacked RV/TLC measurements met the criteria when assessed by the combination of the flat chest index and BMI (Figure 5). When the RV/TLC is not measured, it may be reasonable to use the flat chest index instead for the diagnosis of IPPFE.

IPPFE patients who met the physiological criteria had a significantly lower FVC than those who did not, suggesting that patients with early IPPFE may not meet the physiological criteria. Indeed, half of the IPPFE patients who did not meet the physiological criteria at the time of the diagnosis came to meet the criteria as the disease progressed. Clinicians should be aware that IPPFE patients may not meet the physiologic criteria in their early phase. In addition, it is necessary to evaluate the physiological criteria not only at the time of the diagnosis but also during the clinical course.

Several limitations associated with the present study warrant mentioning. First, this was a retrospective study conducted in a single-center, and the number of patients was relatively small. Therefore, a larger-scale study is necessary to confirm our results. Second, although the sensitivity and specificity of the physiologic criteria of IPPFE in this study were equivalent to those in the study by Watanabe et al. [13], it is difficult to compare the results directly because the study populations were different between the two studies: Watanabe et al. [13] examined pathologically diagnosed IPPFE patients, but we examined only three IPPFE patients who underwent a surgical lung biopsy (definite IPPFE). However, Suzuki et al. [31] showed that there was no marked clinical difference between pathologically diagnosed and clinically diagnosed IPPFE patients. Third, we excluded patients with radiologically possible IPPFE from the study because radiologically possible IPPFE might include apical cap fibrosis other than the early-stage IPPFE, which could create a bias. However, it was not appropriate to include patients with radiologically possible IPPFE in this study because this group may include patients who are not diagnosed with IPPFE. Fourth, the flat chest index is relatively easy to measure on CT images taken in daily practice, some complications of measurement may prevent the flat chest index from being generalized.

In conclusion, we demonstrated the utility and difficulties associated with the physiological diagnostic criteria for IPPFE. We verified the good performance of the physiologic criteria according to the combination of RV/TLC %pred. and BMI in a different cohort. The flat chest index may be used as an alternative physiologic parameter to RV/TLC %pred.

## Figures and Tables

**Table 1:** Clinical characteristics.

Factor	IPPFE (*n* = 37)	IPF (*n* = 89)	*p*-Value
Clinical data			
Age, years	69.6 ± 14.7	74.4 ± 7.23	0.016
Gender, male/female	21/16	73/16	0.006
Brinkman Index	208 ± 331	744 ± 757	<0.001
BMI, kg/m^2^	17.9 ± 3.17	22.8 ± 2.98	<0.001
Flat chest index	0.57 ± 0.07	0.65 ± 0.05	<0.001
Serum KL-6, U/mL	574 ± 345	1100 ± 798	<0.001
Respiratory function parameters			
FVC, %pred.	66.6 ± 23.2	87.4 ± 17.2	<0.001
FEV_1_/FVC, %	90.6 ± 9.82	80.0 ± 8.36	<0.001
FRC, %pred.	78.2 ± 18.2	70.8 ± 18.0	0.072
RV, %pred.	86.9 ± 27.5	64.0 ± 19.7	<0.001
TLC, %pred.	73.9 ± 14.6	76.3 ± 14.3	0.453
RV/TLC, %pred.	124 ± 29.5	86.3 ± 19.2	<0.001
DLco, %pred.	103 ± 49.8	74.6 ± 37.2	0.005

BMI, body mass index; DLco, diffusion capacity of the lung for carbon monoxide; FVC, forced vital capacity; FEV_1_, forced expiratory volume in 1 s; FRC, functional residual capacity; IPF, idiopathic pulmonary fibrosis; KL-6, Krebs von Lungen-6 antigen; RV, residual volume; TLC, total lung capacity. The Brinkman index is calculated as the number of cigarettes smoked per day multiplied by the number of years of smoking.

**Table 2:** Sensitivity, specificity, and area under curve of each parameter for discriminating IPPFE from IPF.

	Present Study				Watanabe et al [13]			
	Cut-Off	Sensitivity	Specificity	AUC	Cut-Off	Sensitivity	Specificity	AUC
BMI, kg/m^2^	20	75.0%	88.0%	0.875	20	78.1%	82.5%	0.881
RV/TLC, %pred.	115	64.3%	93.3%	0.878	115	75.6%	88.7%	0.908
Flat chest index	0.58	61.5%	92.0%	0.837	NA	NA	NA	NA
BMI + RV/TLC		78.6%	88.0%			87.8%	83.5%	
BMI + flat chest index		82.1%	89.3%			NA	NA	

AUC, area under curve; BMI, body mass index; NA, not available; RV, residual volume; TLC, total lung capacity.

**Table 3:** The comparison of the clinical characteristics between IPPFE patients who met the physiological criteria at the time of the diagnosis and those who did not.

Factor	IPPFE with Physiologically Positive (*n* = 22)	IPPFE with Physiologically Negative (*n* = 6)	*p*-Value
Clinical data			
Age, years	72.0 (67.5–75.7)	66.0 (63.7–77.2)	0.736
Gender, male/female	11/11	5/1	0.196
Brinkman Index	0 (0–550)	180 (40.0–650)	0.273
BMI, kg/m^2^	17.4 (14.9–18.8)	21.7 (21.2–21.9)	<0.001
Flat chest index	0.55 (0.50–0.59)	0.65 (0.62–0.69)	0.002
Serum KL-6, U/mL	634 (290–798)	442 (394–573)	0.933
Respiratory function parameters			
FVC, %pred.	58.4 (46.8–72.8)	91.8 (84.1–96.2)	0.008
FEV_1_/FVC, %	94.4 (86.5–100)	87.8 (84.0–90.6)	0.084
FRC, %pred.	76.4 (68.0–90.0)	81.6 (58.2–92.1)	0.867
RV, %pred.	88.8 (69.8–94.4)	81.4 (62.1–84.3)	0.373
TLC, %pred.	69.0 (63.8–81.2)	78.8 (71.0–89.2)	0.314
RV/TLC, %pred.	128 (116–141)	98.0 (90.9–104)	0.002
DLco, %pred.	95.0 (80.6–119)	98.6 (87.2–114)	0.768
CT findings, *n* (%)			
Lower lobe UIP	3 (13.6)	3 (50)	0.091
Lower lobe ILD (including UIP)	11 (50)	5 (83.3)	0.196

BMI, body mass index; DLco, diffusion capacity of the lung for carbon monoxide; FVC, forced vital capacity; FEV1, forced expiratory volume in 1 s; FRC, functional residual capacity; ILD, interstitial lung diseases; KL-6, Krebs von Lungen-6 antigen; RV, residual volume; TLC, total lung capacity; UIP, usual interstitial pneumonia. The Brinkman index is calculated as the number of cigarettes smoked per day multiplied by the number of years of smoking.
